# Can Comprehensive Geriatric Assessment Meet Frail Older People’s Needs? Results from the Randomized Controlled Study CGA-Swed

**DOI:** 10.3390/geriatrics5040101

**Published:** 2020-12-04

**Authors:** Theresa Westgård, Isabelle Andersson Hammar, Synneve Dahlin-Ivanoff, Katarina Wilhelmson

**Affiliations:** 1 Department of Health and Rehabilitation, Institute of Neuroscience and Physiology, The Sahlgrenska Academy, University of Gothenburg, 405 30 Gothenburg, Sweden; isabelle.a-h@neuro.gu.se (I.A.H.); katarina.wilhelmson@gu.se (K.W.); 2 Centre of Aging and Health-AGECAP, University of Gothenburg, 405 30 Gothenburg, Sweden; synneve.dahlin-ivanoff@neuro.gu.se; 3 Department of Psychiatry and Neurochemistry, Institute of Neuroscience and Physiology, The Sahlgrenska Academy, University of Gothenburg, 405 30 Gothenburg, Sweden; 4 Department of Geriatrics, The Sahlgrenska University Hospital, 413 45 Gothenburg, Sweden

**Keywords:** geriatric, frail older people, person-centered approach, intervention

## Abstract

Background: The comprehensive geriatric assessment (CGA) designed to manage frail older people requiring acute medical care, is responsible for diagnostics, assessment, treatment, and planning while addressing a person’s medical, psychological, social, and functional capabilities. The aim was to investigate if CGA had an impact on frail older people’s activities of daily living (ADL) status, self-rated health, and satisfaction with hospital care. Methods: A two-armed design with frail people aged 75 or older who required an unplanned hospital admission were randomized to either the CGA ward or to an acute medical ward. Analyses were made based on the intention-to-treat principle (ITT). The primary outcome was ADL. Data were analyzed using Chi-square and odds ratio. A subgroup analysis was performed due to non-adherence and contamination. Results: One-hundred and fifty-five people participated in the study; 78 in the intervention and 77 in the control. Participants in the intervention group had a higher odds ratio of reporting having received written information and felt that care met their needs during their hospital stay. No additional statistically significant results for the primary or secondary outcomes in the ITT analysis were achieved. Conclusion: Participants felt that the care they received with the CGA ward met their needs. The lack of additional results supporting the CGA could be due to difficulties performing pragmatic intervention trials in clinical hospital settings, and because a CGA during one hospital stay is probably not enough to have long-term effects.

## 1. Introduction

The complex needs of frail older people are often not understood [1] because the current healthcare system is mainly designed to address one medical issue at a time, and because it is not equipped to meet people with multifaceted needs [2]. Frailty is an age-related decline across multiple physiological systems, which results in a state of decreased reserve resistance to stressors [3,4]. Frail older people are at a higher risk of further deterioration if their needs are not acknowledged. Frailty is related to comorbidities and dependence in activities of daily living (ADL) [5,6,7]. It is known that the early stages of the frailty process may be clinically silent, however, when depleted reserves reach a combined threshold leading to serious vulnerability, the syndrome may become detectable by looking at clinical, functional, behavioral, and biological markers [8]. Thus, frail older people are in need of early identification and interventions to prevent further deterioration. As a consequence, people who are frail have a higher prevalence of the risk of falling, hospitalization, and institutionalization [9,10]. Comprehensive geriatric assessment (CGA) is designed to manage these challenges through the coordination of multidimensional diagnostics by an interdisciplinary treatment team working to determine medical, psychological, social, and functional capabilities [11,12,13] of the frail older person resulting in personalized treatment, rehabilitation, and follow-up. To date, CGA remains the most effective intervention for frail older people [14,15,16]. A person-centered approach is recommended as a way to meet the care needs of older adults with comorbidities and dependence in ADL [17] and could be an important part of CGA.

In recent years, the need for an age-friendly health system has been promoted as a movement with the purpose of recruiting the entire healthcare system to focus on the most important areas in delivering quality healthcare to older people [18]. CGA information and planning could be a part of this evidence-based framework and integrates the “4 Ms” securing that health care is age-friendly throughout the health care chain. The first M’s aim, is to ensure that older people will have a mobility plan which entails keeping older people safely moving. The second M’s aim, medication review, is to ensure that people are not receiving medications that negatively impact their mobility or mentation. The third M’s aim, mentation is to identify and understand mental status changes and issues of depression, dementia, and delirium and provide the necessary preventative care, treatment, and long-term management throughout the health care continuum. Finally, the fourth M’s aim is to address what matters, based on knowing and understanding what matters to the older people’s health outcomes goals and care preferences [19].

Additionally, a common element built into the CGA is to assess a person’s ADL status [20], which has been proven to be a powerful and useful indicator of health and functional status [5,6]. Using CGA makes it possible to shift one’s perspective away from a disease- and problem-oriented approach to care. It can support the staff in seeing the geriatric patient as a person and not as a disease [21], which can benefit the complex needs of the frail older patient [1]. Frequently, the functional abilities of daily living and the symptoms related to these decreases are either ignored or underemphasized in older patients [22]. Reuben [7] highlights that we subconsciously take our ability to complete ADL for granted when we are in good health. Yet when the performance of daily activities becomes a struggle, it is a strong signal that decline already has occurred [7].

A recent umbrella review of CGA [20] points out that patient-related outcomes seldom are reported. These outcomes could be measured with self-rated health [23] and satisfaction with ADL, health, and care. The measurement could include a person’s individual consciousness of health status and subjective information that is only known by the person themselves [24]. Consequently, when asking a person to rate their health, an opportunity to increase the understanding of older people’s health status [25] could be achieved.

Even though CGA is seen as a golden standard for frail older people [14,15,16], recent randomized control trials (RCTs) are lacking [26]. Swedish healthcare has undergone dramatic changes and resources have been redistributed, resulting in Sweden having the lowest number of hospital beds per capita in Europe, especially in geriatrics [27,28]. In addition, there is a need for studies evaluating CGA and measuring patient-related outcomes [20].

The aim of this study was to investigate if the CGA had an impact on frail older people’s ADL status, self-rated health, and satisfaction with hospital care after acute hospital admission. To examine this, a two-armed randomized control study was designed with the hypothesis that frail older people who received a CGA would maintain their ADL, self-rated health, and satisfaction with ADL as well as satisfaction with physical and mental health to a higher degree compared to the control group up to six months after an acute hospital admission.

## 2. Materials and Methods

### 2.1. Study Design

The study has a two-armed design with participants randomized into the intervention or the control group in a university hospital. Follow-up after hospital discharge occurred at one, six, and twelve months. All data collection was conducted between March 2016 and January 2020. However, this study’s data analysis was performed prior to the data from the twelve-month follow up being completed, and, therefore, only the data from the baseline, one-, and six-month follow-ups are included in this study.

### 2.2. Ethics Approval and Consent to Participate

Ethical approval was obtained for the study, ref. no: 4 899-15, Regional Ethical Review Board in Gothenburg, Sweden. Trial Registration: Clin.Trial.gov NCT02773914.

### 2.3. Participants and Setting

Eligible for inclusion were frail older people aged 75 or older who came to the emergency department and required an acute medical hospital admission. Exclusion criteria were being admitted via a fast track (for stroke, coronary infarct, or a hip fracture). For more information and details about the study, see the pilot and study protocol [29,30].

### 2.4. Recruitment, Consent, and Randomization

Eligible participants were identified by using the FRESH screening tool [31]. Potential participants were invited to join by the care coordinator in the emergency department. Information was provided both verbally and in writing. Those who agreed to participate signed a consent form. Randomization was carried out with the help of a computer-generated number which then was assigned by the case coordinator. Allocation was concealed by using opaque sealed envelopes that were numbered sequentially.

### 2.5. Intervention Group

The participants who were randomized to the intervention group received CGA on a geriatric acute medical ward. Assessments were both comprehensive and person-centered and were provided by a multidisciplinary team to address the frail older people’s multiple needs related to physical health, functional ability, psychological state, cognition, and social-environmental circumstances [15,30,32]. The CGA team consisted of geriatrically-trained medical doctors, nurses, nurse assistants, occupational therapists (OTs), and physical therapists (PTs). When necessary, the team also included a social worker (SW) and nutritionist. The information and knowledge accumulated by the multidisciplinary team were used to tailor treatment and rehabilitation individually. The follow-up care plans and evaluation of long-term care needs were planned and coordinated. All activities on the intervention ward were carried out in partnership with the frail older person. Furthermore, those responsible for managing care after discharge were informed about care during the hospital stay and planned follow-up, so as to enhance communication and understanding while aiming to optimize the person’s functional status. The multidisciplinary team members had clearly defined domains and assessments in supporting the hospital care provided on the CGA ward and in the planning of discharge. Assessments were administered to ensure a holistic and comprehensive evaluation of the frail older patients’ medical status, self-assessed health, functional status, psychological status, social situation, and environment related to the person’s health status and life situation. The physician was responsible for illness burden, medical review, symptoms, somatic status, pharmaceutical review, self-assessed health, and depression. Nurses were responsible for symptoms, nutritional status, self-assessed health, sight and hearing, social network, formal support, financial support, living conditions, and transports. OTs were responsible for activities of daily living, cognition, and accessibility and assistive devices, while PTs were responsible for physical functioning. When necessary, a dietician was consulted for nutritional support and a social worker was consulted for support concerning social network, formal support, financial support, living conditions, transport, and accessibility.

### 2.6. Control Group

The participants who were randomized to the control group received treatment on the acute medical wards, staffed by a medical doctor, nurse, and nurse assistant. The control group received the usual acute hospital care, i.e., care given at an ordinary medical hospital ward without a specialized multi-disciplinary team practicing the CGA. The acute medical problem and symptoms of the patient determined the assessments and care provided on the control wards. The control ward did not work according to the comprehensive and person-centered approach included in a CGA [30].

Treatments and services such as those provided by OTs, PTs, SWs, and the nutritionists were not automatically included on the acute medical wards; rather, referrals were required from physicians or nursing staff if a participant required additional consultation, assessment, or treatment by these professions.

### 2.7. Data Collection

All of the researchers were licensed health care professionals. Prior to the baseline interviews, the researchers conducted a chart review and then completed the data collection with the participants on their respective wards. Baseline data were predominantly collected on the ward during their acute admission. However, if the patient was transferred to another ward, facility, or home prior to the completion of data collection, rather than losing the participant, they were followed to their new location where the baseline data collection was completed as soon as possible. Since the baseline data were collected during the hospital stay, the intervention group had received parts of the CGA prior to baseline data collection. Follow-ups after one month and six months were carried out in the person’s home or at the place of discharge. Whenever possible, the intention was that the researcher performing the follow-up was blinded to avoid the risk of bias.

### 2.8. Measurement of Frailty

The following measurements and cut-off levels of frailty indicators were used based on Fried’s [33] criteria, with the addition of visual and cognitive impairment because of the high impact on disability. Weakness: Reduced grip strength considered to be below the lowest norm range for ages 80 to 84, 13 kg for women and 21 kg for men for the right hand, and below 10 kg for women and 18 kg for men for the left hand, using a North Coast dynamometer [34]. Fatigue: Question from the Göteborg Quality of Life Instrument [35], answering “yes” to the question “have you suffered any general fatigue/tiredness over the last three months?” Weight loss: Question from the Göteborg Quality of Life Instrument [35], answering “yes” to the question “have you suffered any weight loss over the last three months?” Reduced physical activity: Taking one to two or fewer outdoor walks per week. Impaired balance: The Berg Balance Scale [36], reduced balance is defined as having a value of 47 or less. Reduced gait speed: Walking four meters with a gait speed of 0.6 m/second or slower [37]. Visual impairment: The KM chart [38], impaired vision is defined as having a visual acuity of 0.5 or less. Impaired cognition: The MMSE [39], impaired cognition is defined as having a score below 25. In this study, frailty was categorized into non-frail (zero indicators), pre-frail (one to two indicators), and frail (three or more indicators).

## 3. Outcome Measures and Assessments

### 3.1. Primary Outcome

ADL were measured using the ADL staircase [40]. This assessment was used to measure the independence/dependence on another person in five personal ADL (P-ADL) items (bathing, dressing, going to the toilet, transferring, and feeding) and four instrumental (I-ADL) items (shopping, cooking, cleaning, and transportation). The ADL staircase has been reported to have good reliability and validity [41]. The sum of dependence in the nine ADL was calculated, range zero to nine, with a clinically significant change of one or more units between the baseline and follow-up.

### 3.2. Secondary Outcomes

The secondary outcome measures were self-rated health, questions about satisfaction with ADL, health, and satisfaction concerning hospital care.

Self-rated health was measured by asking people to evaluate their health status based on a single question taken from the SF-36 [42]: “in general how would you say your health is?”, scored with a five-point scale. This question, which is subjective in nature, has responses that are scaled as excellent, very good, good, fair, and poor. A clinically significant change of one or more unit between baseline and follow-up was used.

Three questions from the Fugl-Meyer-Lisat-11 [43], exploring how a person subjectively experiences their physical health status, psychological health status, and ADL, were used to measure how satisfied they were with their health. Participants were provided with a scale with the following responses: very unsatisfied, unsatisfied, somewhat unsatisfied, somewhat satisfied, satisfied, very satisfied. A clinically significant change of one or more unit between baseline and follow-up was used.

Eight hospital care questions based on the Pyramid Questionnaire [44,45] were also included. These questions were only asked at the one-month follow-up and were used to measure older people’s perceptions of quality of care. The questions and statements were as follows: did you receive verbal information about evaluations, care, and treatment during the hospital stay?; did you receive written information about evaluations, care, and treatment during the hospital stay?; I feel that the care I received during my hospital stay meets my needs; I feel that the care planning meeting before discharge was valuable; I was able to take part in the discussion of my needs in the care planning meeting; I feel that the actions planned were equal to my needs (home nursing care, rehabilitation, training, assistive devices, and/or home modifications); I feel that the actions delivered were equal to my needs (home nursing care, rehabilitation, training, assistive devices, and/or home modifications); I am satisfied with the hospital care. To respond to each statement, participants were provided with a scale with the following responses: agree completely, agree partly, neither agree nor disagree, disagree, and disagree completely. The proportion of participants being satisfied (agree completely and agree partly) between the intervention and control groups was compared.

## 4. Sample Size and Power Calculation

Based on the primary outcome variable, dependence in activities of daily living, a power calculation was created (range zero to nine) with an assumed difference between the intervention and control groups of one dependence (i.e., dependent in one more activity of daily living), and a standard deviation of two in both groups. To detect a difference between the intervention and control groups with a two-sided test with a significance level of α = 0.05 and 80% power, a minimum of 64 participants in each group were required. The power calculation and the assumed loss at follow-ups were based on previous research with frail older people [46] and were calculated at 22%. Therefore, a total of 155 people (77 in the control group and 78 in the intervention group) were deemed sufficient to maintain power and were included in the study.

## 5. Statistical Analyses

Analyses were conducted on the basis of the intention-to-treat principle (ITT) whenever possible, meaning that participants are analyzed on the basis of the group to which they initially were randomized. Given that the participants are old and frail, a high drop-out rate was anticipated. For this reason, analyzing only complete cases was not a relevant option as it could lead to bias since missing data would not be random. Therefore, a data imputation approach was used to replace missing values with a value based on the median change of deterioration (MCD) between baseline and follow-ups [47]. The reasons for this imputation method were that (1) the frail older people were expected to deteriorate over time as a natural course of the aging process, and (2) deteriorated health often was the reason for not participating in the follow-ups. The worst-case change was imputed for those who died before follow-up. Sensitivity analyses were also performed to test MCD with complete cases to confirm that the results were aligned [48].

It is known that RCTs are at risk of having some form of contamination [49] and non-adherence [50]. In our study, the chart review in the process evaluation revealed contamination both because some participants in the control group were readmitted to a ward working according to CGA while active as a control, and because of non-adherence in the intervention group as participants did not always receive a full CGA. These errors occurred because (1) treatment contamination occurred with six control participants, four before the one-month and two before the six-month follow-up, because of treatment crossover while they were active as a control; (2) non-adherence for 22 intervention participants, where a chart review revealed that they had not received a full CGA (defined as receiving occupational therapy, discharge planning, and a minimum of three risk assessments); and (3) nursing home residents were not prioritized for assessment by occupational therapists in the hospital, which we became aware of over the course of the study. As a consequence, when removing participants due to non-adherence, the proportion of nursing home residents in the intervention group became much lower than in the control group. We, therefore, decided to remove all nursing home residents in both groups at both follow-ups to maintain comparability between the two groups (two from the intervention and five from the control group). For this reason, all the results will be presented in two tables, one for the intention-to-treat analysis and one for the subgroup analysis (without contamination and non-adherence).

In total, 11 control participants were removed in the subgroup analysis, nine at one month and an additional two at six months. In the intervention group, 24 participants were removed from the subgroup analysis at both follow-ups.

Statistical analyses were conducted using IBM SPSS Statistics for Windows, Version 24.0, 2016, Armonk, NY: IBM Corp. Both descriptive and analytical statistics were used in order to compare groups and to analyze changes over time. The Chi-square was used to test differences in the proportions between the two groups at the baseline. The number of participants that improved, maintained, or decreased their level of ADL and SRH at one month and six months was compared to the baseline. These outcomes were analyzed using Chi-square and odds ratio (OR) to compare the CGA intervention and control groups. Two-sided significance tests were used throughout the analysis, and a value of *p* ≤ 0.05 and a 95% confidence interval (CI) were considered statistically significant.

## 6. Results

**Figure 1:**
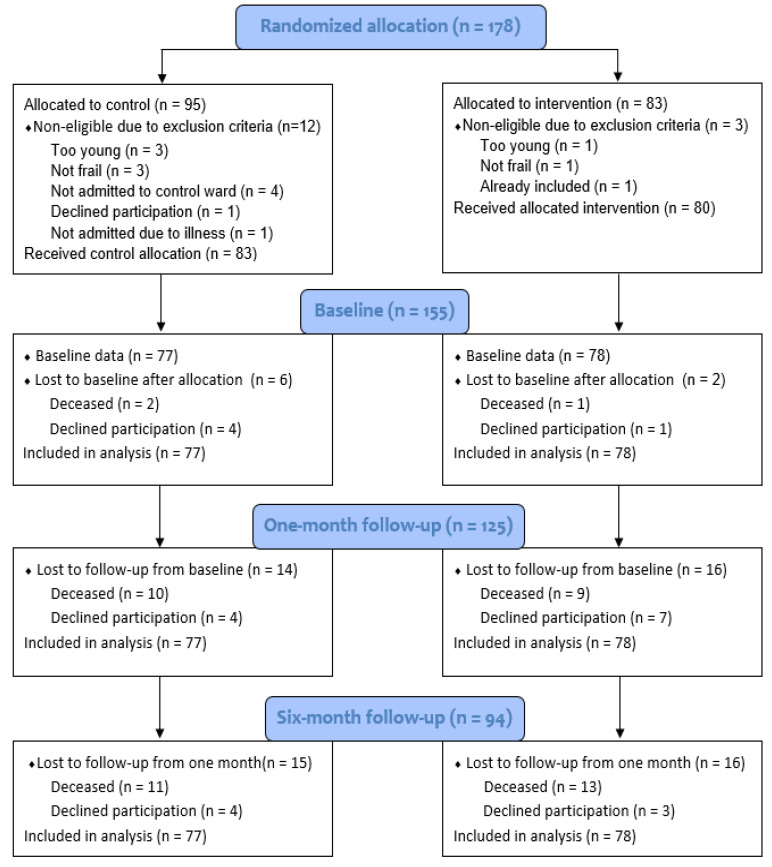
Consort 2010 flow diagram for the CGA RCT.

The flow of the participants throughout the RCT from baseline to six months is shown in the CONSORT diagram, Figure 1. The drop-out rates were similar in the intervention and the control groups at the one-month and six-month follow-ups.

Demographics, clinical characteristics, and illness burden was collected on all participants in the study, shown in Table 1. There were no statistically significant differences between the two groups’ baseline characteristics, and the two groups were equally burdened by illness.

There were no statistically significant differences between the intervention and control groups for the primary outcomes in the intention-to-treat analysis and subgroup analysis, see Table 2 and Table 3. However, for the intervention group, both the intention-to-treat analysis and the subgroup analysis showed a trend towards a higher OR for improved ADL at both follow-ups. Additionally, sensitivity analysis of complete cases for the severity of frailty was examined to test ADL status change and confirmed that the results were aligned (data not shown). Lastly, all baseline frailty indicators were analyzed in the intention to treat groups for their ADL status outcomes during all follow-ups (data not shown). These analyses resulted in one statistically significant result, where those not frail for gait speed at baseline in the intervention group had lower odds (OR 0.15, CI 0.03–0.78) of having decreased ADL status compared to the control group from baseline to six months follow up.

There were no statistically significant differences for any of the secondary outcomes between the intervention and control groups in the intention-to-treat analysis, see Table 4. In the subgroup analysis, there was a lower OR for most secondary outcomes, favoring the control group. One OR for satisfaction with ADL from one month to six months was statistically significant (OR 0.45) for the control group, see Table 5. All secondary outcomes have been analyzed for complete cases, and they showed aligned trends.

Two of the eight hospital care questions that measured participants’ perceptions of quality of care one month after discharge were statistically significant for the intervention group. They reported to a higher degree receiving written information from the hospital (OR 2.19) and that the care received in the hospital met their needs (OR of 2.83), see Table 6. In the subgroup analysis, there was a statistically significant result for the intervention group that hospital care met their needs (OR 4.43), see Table 7.

## 7. Discussion

The trial is reported essentially as a negative result since the CGA intervention showed no statistically significant results for independence in ADL, self-rated health, and satisfaction with ADL, physical, and mental health at all follow-ups, with the exception of one statistically significant result for satisfaction with ADL in favor of the control group. One possible explanation for the lack of statistically significant results could be that CGA has no effect. Another possible explanation could be some participants may have been too frail to benefit from receiving a CGA. The possibility of this explanation could be that participants may have been too frail to benefit from a CGA, however when analyzed for complete cases, there were no statistically significant trends related to the severity of frailty and ADL independence. Despite the lack of outcomes, this study should not necessarily be seen as a failure of a CGA, rather there are several other possible reasons for the negative trial result and lessons were learned that are worth discussing, related to the difficulties associated with performing pragmatic intervention trials in a clinical setting with frail older people. The fact that the intervention group felt that the care given during the hospital stay met their needs to a higher degree compared to the control group might indicate that CGA has an effect.

A recent umbrella study has criticized that levels and aspects of frailty in CGA recipients and patient-related outcomes are usually not reported [20]. However, these findings could be relevant and warrant additional exploration in future studies since previous research has identified that the combination of for example slow gait and cognitive complaints (the Motoric Cognitive Risk syndrome) is a new clinical tool useful in identifying those at high risk of developing dementia and therefore may be used to target interventions [52]. Evidence is limited but cognitive training and targeted physical activity may be useful to lessen or prevent gait and cognitive declines with age [52]. Thus, we analyzed if ADL status was different dependent upon individual frailty indicators in the intention to treat analysis in this study. While the tests were based on the different frailty variables, unfortunately, the small sample sizes and numerous significance tests, made it not feasible to draw any conclusions from these results. Although, they may indicate that CGA has a better effect on those whose level of frailty is low, specifically for gait speed but more research is required. This may be relevant as an earlier study found that being able to describe the clinical profiles of frail patients considered as non-responders to care, could be more likely to rapidly progress for decreased cognitive function, as in the case of Alzheimer’s disease (AD), compared to other patients who have an accumulation of frailty without AD [53]. Additionally, although not statistically significant, the intervention group had a higher tendency of having improved ADL independence at the follow-ups. This result supports in maintaining and/or improving their ADL independence [47]. A CGA team should assess a person’s ADL status [20], as ADL has been proven to be a powerful and useful indicator of functional status and health [5,6,54]. Previous research has shown effects on ADL after receiving a CGA [55], and one reason why our study failed to show significant results on ADL could be low statistical power. Frail older people in need of acute medical care are a heterogeneous group [56] with high comorbidity and mortality [4]. As a consequence, a large sample size is needed and missing data needs to be procedurally managed [57]. Despite using imputation to handle missing data, statistical power might still be too low. Another reason could be that while selection by the frailty of participants for the CGA does work, the ADL measures used to assess the primary outcome were not sufficiently sensitive to change to detect a significance amongst the groups. This is supported by the 2017 Cochrane report which states that CGA probably leads to no or little difference in ADLs [15]. We chose ADLs as our primary outcome prior to the release of Cochrane’s report, and despite the report’s findings, we saw a trend that ADLs, while not statistically significant, supported slightly higher odds of improved ADLs after one and six months with CGA recipients. Furthermore, it is important to highlight that this study is only part of the full RCT, as the long-term effects of the CGA at the one year follow up have yet to be analyzed. This could be relevant, as a previous study exploring frail older people’s results from a continuum of care was positively associated with having higher odds of independence on ADLs at the one year follow up, supporting that there are long-term effects of having an integrated care plan with frail older people [47].

Unfortunately, in our study, there were several disturbances that may have tainted the true results of ADLs after a CGA. This is because in an RCT there are several threats that can occur, which ultimately disturb the validity of the trial [58]. We identified two threats that required action: (1) cross-contamination of the control group, and (2) non-adherence to working according to CGA. The issue of cross-contamination and failure to deliver treatment as intended was difficult to control. During the study, many participants required readmission, and it was not ethical to exclude the controls from being admitted to a ward working according to CGA if needed. This led to contamination of the control group receiving an intervention. The study took much longer than forecasted to complete inclusion and during this time, many staff had quit their posts or changed to work on another ward. In addition, over the course of roughly a six-month period, there was an unusually high work strain on the CGA staff. The ward was forced to restructure and open up ten additional hospital beds within a couple of days due to the lack of available beds at the hospital. As a consequence, new staff were hired quickly, but they did not have the knowledge and experience of working according to CGA. This resulted in periods of time where the intervention was not being carried out as planned at the start of the study. An earlier study supports that the more complicated and different the protocol is from the normal routine, the more likely staff are to wander back to familiar and usual work practices, regardless of their motivation to comply [58]. This human factor was observed, and staff did not always work according to the CGA. The authors have been forthcoming in presenting both the intention-to-treat and subgroup analysis [59], while remaining mindful that the intention-to-treat data is flawed due to contamination and that the subgroup analysis lacks statistical power.

In this study, we examined ADL based on the participant’s satisfaction with their ability to perform their ADL. Our results supported that the intervention group had higher odds of maintaining independence in ADL over time, while they were less satisfied with their ADL status compared with the control group, however. One explanation for the intervention group’s diminished satisfaction concerning their ADL compared to the control group at the follow-ups could be that while they were receiving care on a CGA ward, it also meant they were assessed through multiple measures and tests. As a result, they could have become more aware of their condition and may have felt stigmatized. Screenings have been identified as potentially harmful and resulting in stigma and/or anxiety [60,61]. Mudge et al. [62] found that being identified as frail could signify that a person has failed. Frail older people’s experiences are influenced by whether or not they think they are old and frail, and only they themselves can make the distinction between being labeled as frail and actually feeling frail [63]. However, screening programs are also said to be better than the cure as they lead to an early diagnosis [61]. Since the aim of CGA is to identify needs early, it could have an effect similar to screenings. This is supported by the fact that the intervention group in our study reported that the hospital care they received on the CGA ward met their needs to a statistically significant higher degree than the control group.

Previous studies support that older people often have fragmented care after discharge from the hospital [64,65]. A CGA should be carried over into the person’s personal living environment to secure the best scenario in safeguarding that the care plan is properly followed and implemented according to the person’s wants and wishes. A CGA might have an expiry date and require updates since older people have long-term needs. Reuben et al. [66] reported in their conclusion following a CGA RCT that a CGA team performing one-time frailty assessments cannot improve functional status or affect mortality for hospitalized patients. Rather, they underscored that frail, older people who have been hospitalized would best benefit from a CGA that is continuously managed after discharge. This is known as integrated care, which would be best achieved by a multidisciplinary team [67]. Dahlin-Ivanoff et al. [68] explained that frail older people differ and that they can be frail in a variety of ways, which for some can fluctuate from day to day or week to week. As a result, the frail person’s abilities can be over- or underestimated, and implications for the assessments, results, and care plans may not be optimal as a result of the fluctuations in frailty [68]. Thus, to best support the frail person after discharge, a collaboration between the CGA team, those receiving the care, and those providing the care to the frail older person after the hospital stay, must be organized and adapted to meet the person’s individual needs in their own environment [68]. Research shows that integrated care has a positive impact on frail older people’s health and functional status [47]. The age-friendly health system [19] could be a framework to support achieving integrated care when the older people’s mobility, medication, mediation, and what matters to them is the focus.

## 8. Conclusions

The trial is reported essentially as a negative result, however, the outcome should not necessarily be seen as a failure of a CGA, as one statistically significant intervention effect in this study was that frail older people felt that the care they received on the CGA ward met their needs, which could indicate a higher quality of care. The lack of additional results supporting the CGA could be due to difficulties performing pragmatic intervention trials in clinical hospital settings leading to a risk of low statistical power. In addition, a CGA during a single hospital stay is probably not enough to have long-term effects, since frail older people are in need of integrated care provided by multidisciplinary teams.

## Figures and Tables

**Table 1:** Baseline characteristics of the participants.

Characteristics	Control Group (n = 77)		Intervention Group (n = 78)		*p*-Value
Age, mean (range)	86.2	(76–98)	87.5	(75–101)	0.17
MMT ^1^, mean (range)	24.3	(13–30)	23.0	(9–30)	0.14
	n	(%)	n	(%)	
Female	43	(55.8)	47	(60.3)	0.58
Living alone	48	(62.3)	51	(65.5)	0.70
Tertiary education ^2^	16	(20.1)	8	(10.3)	0.404
Independent in ADL	5	(6.5)	4	(5.1)	0.747
CIRS–G ^3^ ≥ 3 in any category, %	72	(93.5)	77	(98.7)	0.26
CIRS-G ^3^ median number of ratings 3–4 (range)		3 (0–9)		3 (0–7)	
Good self-rated health ^4^	21	(27.3)	26	(33.8)	0.41
0 frailty indicators (non-frail) ^5^	0	(0)	0	(0)	-
1 frailty indicator (pre-frail) ^5^	1	(1.2)	0	(0)	-
2 frailty indicators (pre-frail) ^5^	5	(6.5)	3	(3.8)	0.461
3 frailty indicators (frail)	9	(11.9)	6	(7.7)	0.403
4 frailty indicators (frail)	10	(13)	12	(15.4)	0.669
5 frailty indicators (frail)	14	(18.2)	17	(21.8)	0.574
6 frailty indicators (frail)	16	(20.8)	20	(25.7)	0.474
7 frailty indicators (frail)	15	(19.5)	14	(17.9)	0.807
8 frailty indicators (frail)	7	(9.1)	6	(7.7)	0.754
Satisfaction with ADL ^6^	32	(41.6)	26	(33.3)	0.291
Satisfaction with physical health ^6^	7	(9)	11	(14.1)	0.334
Satisfaction with mental health ^6^	31	(40.3)	25	(32.0)	0.288

^1^ MMT control n = 74, intervention n = 73; ^2^ tertiary education (partial or completed university or college); ^3^ CIRS–G = Cumulative Illness Rating Scale-Geriatric [51], 3 = severe/constant significant disability/”uncontrollable” chronic problems or conditions, 4 = extremely severe/immediate treatment required/end-organ failure/severe impairment in function. ^4^ SRH = self-rated health (excellent/very good/good), one person missing from intervention group SRH baseline data; ^5^ frailty measured with the following frailty indicators: weakness, fatigue, weight loss, physical activity, poor balance, slow gait speed, visual impairments, and cognition. Categorized into non-frail (0 indicators), pre-frail (1–2 indicators), and frail (≥3 indicators); ^6^ Satisfaction = very satisfied, satisfied.

**Table 2:** Changes in levels of activities of daily living (ADL) at follow-ups. Intention to treat; odds ratio (OR) with 95% confidence interval (CI).

Change in ADL	Control Group Baseline (n = 77)		OR	Intervention Group Baseline (n = 78)			*p*-Value	OR
	n	(%)		n	(%)	(95% CI)		
**Improved ADL**								
Baseline to one month	21	27.3	1	28	35.9	0.75–2.95	0.249	1.49
Baseline to six months	18	23.4	1	21	26.7	0.58–2.50	0.611	1.21
One month to six months	14	18.2	1	18	23.4	0.62–2.95	0.452	1.35
**Maintained levels of ADL**								
Baseline to one month	21	27.3	1	20	26.0	0.45–1.88	0.818	0.92
Baseline to six months	15	19.5	1	18	23.1	0.57–2.68	0.585	1.24
One month to six months	33	42.9	1	30	38.5	0.44–1.58	0.578	0.83
**Decreased levels of ADL**								
Baseline to one month	34	44.2	1	30	38.5	0.42–1.50	0.472	0.79
Baseline to six months	44	57.1	1	39	50.0	0.40–1.41	0.373	0.75
One month to six months	30	39.0	1	28	35.9	0.46–1.68	0.694	0.88

**Table 3:** Changes in levels of activities of daily living (ADL) at follow-ups. Subgroup analysis *; odds ratio (OR) with 95% confidence interval (CI); control: baseline to one month n = 68, baseline/one month to six months n = 66. Intervention: n = 54.

Change in ADL	Control Group Baseline (n = 72)		OR	Intervention Group Baseline (n = 54)			*p*-Value	OR
	n	(%)		n	(%)	(95% CI)		
**Improved ADL**								
Baseline to one month	20	29.4	1	18	33.3	0.60–2.80	0.502	1.30
Baseline to six months	17	25.8	1	16	29.1	0.53–2.64	0.682	1.18
One month to six months	12	17.6	1	12	21.8	0.53–3.18	0.562	1.30
**Maintained levels of ADL**								
Baseline to one month	19	27.9	1	13	23.6	0.39–2.00	0.768	0.88
Baseline to six months	13	19.1	1	9	16.4	0.31–2.04	0.636	0.80
One month to six months	27	40.9	1	19	34.5	0.36–1.60	0.473	0.76
**Decreased levels of ADL**								
Baseline to one month	29	42.6	1	23	41.8	0.54–2.25	0.794	1.10
Baseline to six months	36	54.5	1	30	54.5	0.49–2.05	1.000	1.00
One month to six months	27	40.9	1	24	43.6	0.54–2.31	0.762	1.12

**Table 4:** Secondary outcomes of self-rated health and satisfaction maintained/improved at follow-ups compared to baseline. Intention to treat; odds ratio (OR) with 95% confidence interval (CI).

Change in Outcome	Control Group Baseline (n = 77)		OR	Intervention Group Baseline (n = 78)			*p*-Value	OR
	n	(%)		n	(%)	(95% CI)		
**Self-Rated Health**								
Baseline to one month	51	66.2	1	48	72.3	0.42–1.58	0.537	0.81
Baseline to six months	49	63.6	1	41	52.6	0.34–1.24	0.192	0.65
One month to six months	52	67.5	1	55	70.5	0.60–2.39	0.600	1.20
**Satisfaction with ADL**								
Baseline to one month	34	57.6	1	26	52.0	0.37–1.70	0.556	0.80
Baseline to six months	31	53.4	1	23	46.0	0.35–1.58	0.441	0.74
One month to six months	45	63.4	1	35	52.2	0.32–1.25	0.186	0.63
**Satisfaction with Physical Health**								
Baseline to one month	35	60.3	1	23	46.9	0.27–1.25	0.167	0.58
Baseline to six months	28	48.3	1	26	53.1	0.57–2.59	0.622	1.21
One month to six months	43	59.7	1	35	53.8	0.49–2.61	0.781	1.13
**Satisfaction with Mental Health**								
Baseline to one month	33	57.9	1	25	50.0	0.34–1.56	0.414	0.73
Baseline to six months	26	45.6	1	25	50.0	0.56–2.55	0.651	1.19
One month to six months	49	68.1	1	42	61.8	0.38–1.52	0.436	0.76

**Table 5:** Secondary outcomes of self-rated health and satisfaction maintained/improved at follow-ups compared to baseline. Subgroup analysis; odds ratio (OR) with 95% confidence interval (CI); control: baseline to one month n = 68, baseline/one month to six months n = 66. Intervention: n = 54.

Change in Outcome	Control Group		OR	Intervention Group			*p*-Value	OR
	n	(%)		n	(%)	(95% CI)		
**Self-Rated Health**								
Baseline to one month	43 ^1^	64.2	1	33 ^1^	62.3	0.44–1.94	0.829	0.94
Baseline to six months	41 ^1^	61.2	1	28 ^1^	51.8	0.30–1.31	0.218	0.63
One month to six months	45	66.2	1	39 ^1^	72.2	0.55–2.67	0.631	1.21
**Satisfaction with ADL**								
Baseline to one month	33 ^2^	60.4	1	18 ^5^	48.6	0.25–1.34	0.201	0.57
Baseline to six months	28 ^3^	53.8	1	14 ^5^	37.8	0.22–1.23	0.138	0.52
One month to six months	40 ^4^	65.6	1	23 ^4^	46.0	0.21–0.96	0.040	0.45
**Satisfaction with Physical Health**								
Baseline to one month	32 ^2^	60.4	1	15 ^6^	41.7	0.20–1.11	0.085	0.47
Baseline to six months	27 ^3^	51.9	1	15 ^7^	41.7	0.28–1.56	0.345	0.66
One month to six months	37 ^4^	60.7	1	25 ^8^	52.1	0.36–2.33	0.863	0.92
**Satisfaction with Mental Health**								
Baseline to one month	30 ^3^	57.7	1	16 ^5^	43.2	0.24–1.31	0.180	0.56
Baseline to six months	25 ^2^	49.0	1	17 ^5^	45.9	0.38–2.06	0.776	0.88
One month to six months	44 ^4^	72.1	1	33 ^9^	64.7	0.32–1.58	0.399	0.71

^1^ one missing, ^2^ fifteen missing, ^3^ fourteen missing ^4^ five missing, ^5^ seventeen missing, ^6^ eighteen missing, ^7^ nineteen missing, ^8^ seven missing, ^9^ four missing.

**Table 6:** Satisfaction * with hospital care. Complete cases.

Hospital Care Question	Control Group			Intervention Group				
	n	(%)	OR ^1^	n	(%)	(95% CI)	*p*-Value	OR
Did you receive verbal information about evaluations, care, and treatment during the hospital stay? C (n = 59) I (n = 57)	26	44	1	34	60	0.90–3.92	0.095	1.88
Did you receive written information about evaluations, care, and treatment during the hospital stay? C (n = 57) I (n = 57)	23	40	1	34	60	1.03–4.62	0.041	2.19
I feel that the care I received during my hospital stay meets my needs C (n = 59) I (n = 56)	42	71	1	49	88	1.07–7.49	0.036	2.83
I feel that the care planning meeting before discharge was valuable C (n = 58) I (n = 57)	32	55	1	33	58	0.53–2.34	0.768	1.12
I was able to take part in the discussion of my needs in the care–planning meeting C (n = 57) I (n = 56)	24	57	1	22	39	0.42–1.89	0.760	0.89
I feel that the actions planned equal my needs C (n = 56) I (n = 56)	40	71	1	37	66	0.35–1.74	0.541	0.78
I feel that the actions delivered equal my needs C (n = 58) I (n = 46)	44	76	1	45	80	0.53–3.18	0.563	1.30
I am satisfied with the hospital care C (n = 58) I (n = 55)	49	84	1	46	84	0.34–2.57	0.902	0.94

^1^ reference, participants in the control group; * satisfaction = totally agree, partly agree.

**Table 7:** Satisfaction * with hospital care at the one-month follow-up. Subgroup analysis.

Hospital Care Question	Control Groups			Intervention Group				
	n	(%)	OR ^1^	n	(%)	(95% CI)	*p*-Value	OR
Did you receive verbal information about evaluations, care, and treatment during the hospital stay? C (n = 53) I (n = 40)	25	47	1	23	58	0.66–3.46	0.325	1.52
Did you receive written information about evaluations, care, and treatment during the hospital stay? C (n = 52) I (n = 40)	23	44	1	19	48	0.50–2.61	0.755	1.14
I feel that the care I received during my hospital stay meets my needs C (n = 53) I (n = 39)	39	74	1	36	92	1.14–16.23	0.031	4.31
I feel that the care planning meeting before discharge was valuable C (n = 53) I (n = 40)	30	57	1	25	63	0.55–2.96	0.567	1.28
I was able to take part in the discussion of my needs in the care-planning meeting C (n = 52) I (n = 39)	23	44	1	16	41	0.38–2.03	0.760	0.88
I feel that the actions planned equal my needs C (n = 51) I (n = 39)	38	44	1	27	69	0.30–1.94	0.580	0.77
I feel that the actions delivered equal my needs C (n = 53) I (n = 39)	41	77	1	31	80	0.41–3.11	0.807	1.13
I am satisfied with the hospital care C (n = 52) I (n = 38)	45	87	1	33	87	0.30–3.52	0.967	1.03

^1^ reference, participants in the control group; * satisfaction = totally agree, partly agree.
